# Sp1 promotes tumour progression by remodelling the mitochondrial network in cervical cancer

**DOI:** 10.1186/s12967-023-04141-3

**Published:** 2023-05-06

**Authors:** Xu Xu, Xiaona Wang, Qihui Chen, Aman Zheng, Donglu Li, Ziqi Meng, Xinran Li, Hanchen Cai, Wangzhi Li, Shiyuan Huang, Fan Wang

**Affiliations:** 1 Department of Obstetrics and Gynecology, The Second Affiliated Hospital and Yuying Children’s Hospital of Wenzhou Medical University, 109 West Xueyuan Road, Lucheng District, Wenzhou City, 325000 Zhejiang Province China; 2 Department of Neurological Rehabilitation, The Second Affiliated Hospital and Yuying Children’s Hospital of Wenzhou Medical University, 109 West Xueyuan Road, Lucheng District, Wenzhou City, 325000 Zhejiang Province China; 3 The Second Clinical Medical College of Wenzhou Medical University, Wenzhou City, 325000 Zhejiang Province China; 4 School of Stomatology, Wenzhou Medical University, Wenzhou City, 325000 Zhejiang Province China

**Keywords:** Specificity protein 1 (Sp1), Mitochondrial network, Glycolysis, Cervical cancer

## Abstract

**Background:**

Cervical cancer remains one of the most prevalent cancers worldwide. Accumulating evidence suggests that specificity protein 1 (Sp1) plays a pivotal role in tumour progression. The underlying role and mechanism of Sp1 in tumour progression remain unclear.

**Methods:**

The protein level of Sp1 in tumour tissues was determined by immunohistochemistry. The effect of Sp1 expression on the biological characteristics of cervical cancer cells was assessed by colony, wound healing, transwell formation, EdU, and TUNEL assays. Finally, the underlying mechanisms and effects of Sp1 on the mitochondrial network and metabolism of cervical cancer were analysed both in vitro and in vivo.

**Results:**

Sp1 expression was upregulated in cervical cancer. Sp1 knockdown suppressed cell proliferation both in vitro and in vivo, while overexpression of Sp1 had the opposite effects. Mechanistically, Sp1 facilitated mitochondrial remodelling by regulating mitofusin 1/2 (Mfn1/2), OPA1 mitochondrial dynamin-like GTPase (Opa1), and dynamin 1-like (Drp1). Additionally, the Sp1-mediated reprogramming of glucose metabolism played a critical role in the progression of cervical cancer cells.

**Conclusions:**

Our study demonstrates that Sp1 plays a vital role in cervical tumorigenesis by regulating the mitochondrial network and reprogramming glucose metabolism. Targeting Sp1 could be an effective strategy for the treatment of cervical cancer.

**Supplementary Information:**

The online version contains supplementary material available at 10.1186/s12967-023-04141-3.

## Background

Cervical cancer is one of the most common diseases in women worldwide, with approximately 500,000 new cases diagnosed and 30,0000 patients dying from this disease in 2018 [1, 2]. In recent years, the incidence of cervical cancer has increased 3% in young adults and adolescents [3]. Despite relatively good control in many high-income countries, it remains the leading cause of cancer-related death in less developed countries [2]. In addition to surgery, screening genes and targeting the ones that are highly associated with cervical cancer development is an important research area for developing anti-cancer drugs.

To support the high proliferation rate of cancer cells, metabolic reprogramming characterized by enhanced aerobic glycolysis, which is also known as the Warburg effect [4], is widely observed in a variety of different cancers, including cervical cancer [5–7]. Mitochondria are dynamic organelles undergoing continuous fusion and fission, which is also defined as mitochondrial dynamics [8]. Mechanistically, outer mitochondrial membrane fusion is mediated by mitofusin 1/2 (Mfn1/2), and inner mitochondrial membrane fusion is mediated by OPA1 mitochondrial dynamin-like GTPase (Opa1) [9]. However, dynamin 1-like (Drp1) wraps around mitochondria and promotes the scission of mitochondrial membranes, which ultimately induces mitochondrial fission [9]. Mitochondrial dynamics are involved in the regulation of metabolic reprogramming in tumour cells. For example, an increase in mitochondrial fusion promoted the production of ATP; however, an increase in mitochondrial fission could induce mitochondrial membrane potential depolarization, resulting in a reduction in mitochondrial energy production [10]. Moreover, in Mfn1 and Mfn2 knockout tumour cells, the mitochondrial respiratory chain was disrupted, and the energy supply was impaired [11]. In addition, Opa1-driven mitochondrial cristae remodelling is also required to regulate ATP production in different cells [12]. More importantly, dysregulated mitochondrial dynamics could induce metabolic reprogramming of cancer cells [13–16]. Jackson et al. showed that alterations in the mitochondrial fusion-fission mechanism promoted aerobic glycolysis [16]. However, the underlying mechanisms of how mitochondrial dynamics are regulated in cervical cancer cells remain unclear.

Transcription factor specificity protein 1 (Sp1) is a member of the SP/KLF (SP/Krüppel-like factor, KLF) family [17]. Previous studies have shown that Sp1 is overexpressed in multiple types of cancers, including cervical cancer [18]. A poor prognosis is associated with high expression of Sp1 in some types of cancer, such as glioblastoma [19], lung cancer [20] and breast cancer [21]. Sp1 is also reported to induce metabolic reprogramming and promote tumorigenesis in several types of cancers. Zhang et al. showed that the ERK/c-Myc pathway enhances the expression of glycolysis-related proteins and induces glucose metabolism reprogramming in leukaemia stem cells by activating Sp1 [22]. Xia et al. showed that Sp1 could promote glycolysis by activating CD147 [23]. Ning et al. found that Sp1 could bind to a specific region of the SNHG22 promoter, thereby promoting glycolysis and cell proliferation in ovarian cancer cells [24]. Accumulating evidence suggests that Sp1 contributes to the regulation of mitochondrial dynamics. For example, Sp1 is involved in the activation of mitochondrial fission by promoting drp1 expression in a diabetic renal tubular injury model [25]. Another study showed that Sp1 induces mitochondrial fusion by promoting Mfn2 expression in HeLa cells [26]. Despite the potential link between Sp1 and mitochondrial dynamics, the intrinsic regulatory relationships among Sp1, mitochondrial dynamics, and metabolic reprogramming in cervical cancer cells remain unclear.

In the present study, we detected Sp1 expression in different types of cervical cancer and assessed the effect of Sp1 on tumour progression. Mechanical studies showed that Sp1 promoted mitochondrial network remodelling and metabolic reprogramming. We suggest that Sp1-mediated mitochondrial network remodelling and glucose metabolism reprogramming are pivotal in cervical cancer progression and can be targeted for cervical carcinoma therapy.

## Materials and methods

### Tissue samples and cell lines

In this study, we collected 60 pairs of cervical carcinoma tumour tissues (40 with squamous carcinoma and 20 with adenocarcinoma) and adjacent nontumor tissues. Protocols were approved by the Ethics Committee of the Second Affiliated Hospital and Yuying Children’s Hospital of Wenzhou Medical University and followed the relevant guidelines and regulations. In addition, cervical carcinoma cells (SiHa and HeLa) were purchased from ATCC (American-type culture collection, Rockville, MD, USA). Cell lines were grown in Dulbecco’s modified Eagle’s medium ((DMEM, Gibco, USA) containing D-glucose (4.5 g/L) and L-glutamine (0.584 g/L) with 10% FBS (fetal bovine serum, Gibco, USA). Cells were cultured in an incubator at 37 °C with 5% CO_2_. The cells were passaged when their growth reached 90% confluence. After washing three times with PBS, 0.25% Trypsin-0.04% EDTA was added to cover the cell surface for 1–2 min. When the cells were fully digested, digestion was terminated by adding the complete medium. After centrifugation, the supernatant was removed and the cells were seeded equally into Petri dishes.

### Immunohistochemistry (IHC) and scores

We conducted IHC experiments following the standard IHC kit instructions (ZSJQB Co., Ltd. China). The dilutions of primary antibodies against Sp1 (ab124804, Abcam) and Ki-67 (ab279653, Abcam) were 1:100 and 1:200, respectively. The IHC score was evaluated by two pathologists independently by light microscopy. The evaluation was scored semiquantitatively as intensity score × percentage score. The staining intensity of the two markers was scored as 0 (negative), 1 (weak), 2 (moderate), and 3 (strong).

### Western blotting

Cell and fresh tissue samples were lysed for 10–20 min on ice using RIPA buffer (Beyotime, China) supplemented with a cocktail of protease inhibitors. Samples were collected and fully lysed by ultrasonic fragmentation. Protein concentrations were determined using BCA Protein Assay reagent (Beyotime, China) according to the manufacturer’s instructions. Proteins from lysed cells were separated by SDS‒PAGE and then transferred to 0.22 or 0.45 μm PVDF membranes (Millipore, USA). The membranes were incubated at 4 °C overnight with specific primary antibodies and then at room temperature for 2 h with HRP-conjugated secondary antibody. The primary antibodies were as follows: Sp1 (1:2000, ab124804, Abcam), MFN 1 (1:10,000, ab129154, Abcam), MFN2 (1:1500, ab56889, Abcam), DRP1 (1:2000, ab184247, Abcam), OPA1 (1:3000, 80,471, Cell Signaling Technology), Bcl2 (1:1000, ab182858, Abcam), Bax (1:5000, ab32503, Abcam), Caspase3 (1:4000, ab13847, Abcam), Cleaved caspase3 (1:1500, 9664, Cell Signaling Technology), PKM (1:5000, ab150377, Abcam), HK2 (1:1000, ab209847, Abcam), GLUT1 (1:5000, ab115730, Abcam), LDHA (1:4000, ab52488, Abcam), and β-actin (1:4000, 20,536–1-AP, Proteintech). The results were detected via enhanced chemiluminescence (ECL) and analysed by ImageJ.

### Transfection with shRNA and siRNA

The Sp1 siRNA and negative control siRNA (Guangzhou RiboBio Co., Ltd. Guangzhou, China) were transfected with Lipofectamine (Thermo Fisher Scientific, MA, USA) following the manufacturer’s protocols. Cells were transfected with siRNA at a concentration of 100 nM. The oligonucleotide sequences of siSp1 and negative control siRNA were sense, 5′-CCAGCTTGGTATCATCACA-3′ and sense, 5′-GGCTCTAGAAAAGCCTATGC-3′, respectively. The lentiviruses [Sp1 overexpression (Ubi-Sp1-3FLAG-gcGFP-puromycin), control of Sp1 overexpression (Ubi-3FLAG-gcGFP-puromycin), Sp1 knockdown (hU6-Sp1-shRNA-gcGFP-puromycin) and its negative control (hU6-gcGFP-puromycin)] were obtained from Shanghai GeneChem Co., Ltd. The sequences of the shRNAs were as follows: Sp1 knockdown shRNA (sense: GCTGGTGGTGATGGAATACAT); and Sp1 negative control (sense: TTCTCCGAACGTGTCACGT). Cells were transfected with a multiplicity of infection (MOI) of 10 and incubated for 1 day. After two days of transfection, puromycin (2 μg/mL) was added to the cell lines to obtain stable cell lines.

### Cell activity detection

A cell counting kit-8 (CCK-8) was used to detect the toxic effect of mithramycin A (GlpBio, Montclair, CA) on human cervical carcinoma cells. Cells were first seeded into 96-well plates in triplicate and treated with different doses of mithramycin A for 12, 24, 36, 48, and 72 h. CCK-8 reagent (NCM Biotech, Suzhou, China) was added to each well for 2 h at 37 °C. A spectrophotometer was used to estimate the optical density at a wavelength of 450 nm.

### Colony formation assay

Cells were plated in six-well plates (200/well) and incubated for 2 weeks with DMEM containing 10% FBS at 37 °C and 5% CO2. After washing three times with PBS, the cells were fixed with paraformaldehyde (4%) for 30 min. Finally, the cell colonies were stained with crystal violet (0.1%) for 10 min.

### Transwell and wound healing assay

The migration and invasion of cells were detected via Transwell chambers (Corning, USA). The cells were plated in serum-free DMEM in the top chamber and with 10% FBS medium in the bottom chamber in 24-well plates for the migration assay. For the invasion assay, the top compartments were filled with Matrigel (Corning, USA) instead. After 24 h, the chambers were washed with PBS twice, fixed with paraformaldehyde (PFA, 4%) for 30 min, and then stained with crystal violet (0.1%). An inverted routine microscope (Nikon Instruments Inc.) was used to count cells in five random fields. For the wound healing assay**,** we scratched a wound when cells reached 90% confluency and observed the area at 0 and 24 h.

### TUNEL assay

Terminal deoxynucleotidyl transferase-mediated dUTP nick end-labelling (TUNEL) was conducted to detect apoptotic cells in cell lines and tissues. The experiment was performed using an In Situ Cell Death Detection Kit (Roche) following the manufacturer’s instructions. The sections were deparaffinized, rehydrated, permeabilized, and then exposed to TUNEL reaction mixture in the dark for 1 h. After DAPI counterstaining, the sections were observed under fluorescence microscopy. The average number of positive cells in three images from each group was determined.

### EdU proliferation assay

We detected cell proliferation using an EdU cell proliferation assay kit (Guangzhou RiboBio Co., Ltd. Guangzhou, China). Cells transfected with Sp1 siRNA and controls were seeded in a 12-well plate and incubated with EdU (50 μM) for 2 h. Then, the cells were subjected to fixation, permeabilization, EdU, and DAPI staining.

### Immunocytochemistry

Cells were fixed in PFA (4%), permeabilized with Triton X-100 (0.1%), blocked in goat serum (10%) for 30 min, incubated with Tomm70A (1:200, 14528-1-AP, Proteintech) at 4 °C overnight, and finally exposed to coralite 594-conjugated goat anti-rabbit immunoglobulin G (IgG) for 1 h.

### Mitochondrial membrane potential (MMP) detection

Tetramethylrhodamine ethyl ester perchlorate (TMRE) was used to analyse MMP. The TMRE dye (Thermo Fisher Scientific, MA, USA) was diluted with DMSO (Solarbio, China) to a final concentration of 200 nM. After various treatments, the cells were collected, washed, resuspended in the culture medium, and stained with TMRM at room temperature for 30 min. The TMRE dye was removed, then washed with PBS. Fluorescence was monitored and imaged with fluorescence microscopy.

### Mitochondrial morphology analysis

MitoTracker staining was used to observe mitochondrial morphology via confocal laser scanning microscopy (CLSM, Leica TCS SP8). Cells were plated into 35 mm confocal dishes and incubated with MitoTracker green FM (M7514, Invitrogen, USA) for 30 min at 37 °C.

### Electron microscopy

Cells were fixed with glutaraldehyde (2.5%), immobilized in osmic acid (1%, O5500, Sigma‒Aldrich), dehydrated with acetone, embedded in Araldite CY 212 (E009, TAAB), and stained with alcoholic uranyl acetate and alkaline lead citrate. The sections were observed under a JEM 1200EX transmission electron microscope (JEOL Ltd, Tokyo, Japan).

### Glucose levels and lactate production assay

Cells with different levels of Sp1 expression were seeded into 6-well plates (6 × 10^5^/well) and collected after 48 h. The levels of glucose and lactate were detected as the total cellular protein concentration by a glucose kit (glucose oxidase method) and lactic acid assay kit (NJJCB Co., Ltd. China), respectively.

### In vivo subcutaneous xenograft models

HeLa cells with genetic alterations (2 × 10^6^ in 0.1 mL of PBS per mouse) were injected subcutaneously into female nude mice (five-week-old, BALA/c-nu, Beijing Weitonglihua Sciences Co. Inc., China), respectively. Mice were divided into 4 groups (3/group): the Sp1-overexpression group, Sp1-knockout group, and respective control groups. The growth of the tumours was monitored twice a week. After 35 days, all the mice were euthanized, and the xenograft tumours were obtained. The volume of the tumour was calculated by the formula: V = (length × width^2^)/2. Finally, the formaldehyde xenograft tissues were dehydrated and paraffin-embedded for sectioning (4 μm). The experiment was approved by the Laboratory Animal Ethics Committee and Laboratory Animal Centre of Wenzhou Medical University.

### RNA sequencing and bioinformatics analysis.

Total RNA was extracted from cells using TRIzol (15596018, Thermo Fisher, USA). RNA sequencing was performed at Guangzhou RiboBio Co. Ltd. Briefly, the mRNA was enriched by oligo (dT) and then fragmented. Purified library products were evaluated, and the libraries were sequenced by Illumina (Illumina, USA). We used DESeq2 to perform gene differential expression analysis. Genes with an adjusted P < 0.05 and a log2 (fold change) > 1 were considered positively differentially expressed genes. The pathway enrichment analyses for GO and KEGG were performed in R. The expression relationships between Sp1 and Mfn1, Mfn2, Opa1, and Drp1 were analysed by the TIMER 2.0 [27] database.

### Statistical analysis

Data are shown as the mean ± SD. Data between groups were compared with Student’s t test or one-way ANOVA according to the condition. Correlations between measured variables were assessed by Spearman’s rank correlation. We used the Kaplan–Meier method to estimate the survival probability and compared it with the log-rank test. Differences with a *P* value < 0.05 were considered statistically significant. All the statistical tests were 2-sided. All analyses were performed using the PRISM 8.0 software.

## Results

### Sp1 was overexpressed in human cervical cancer tissues

**Fig. 1:**
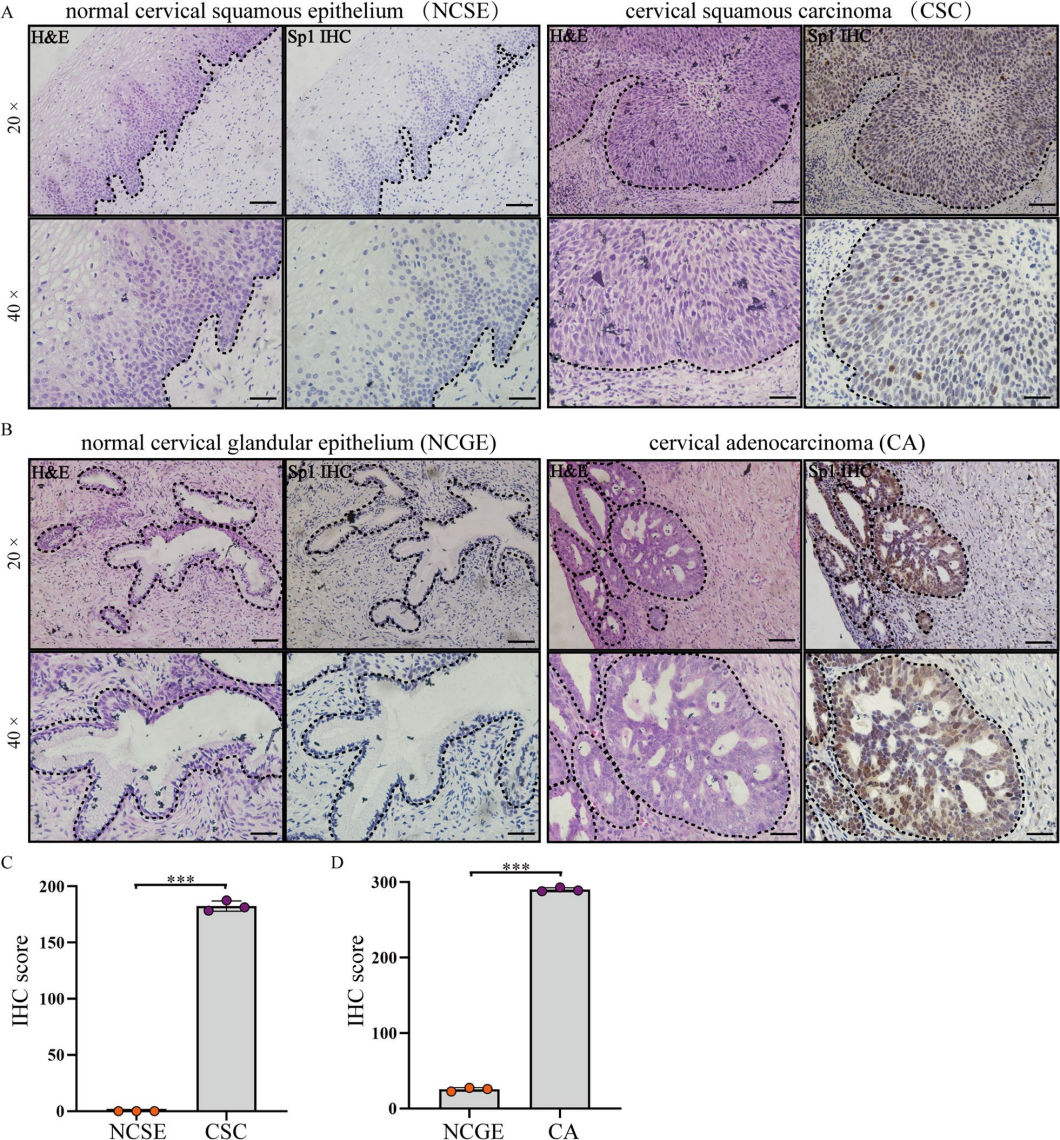
Sp1 was overexpressed in cervical cancer tissues. The expression of Sp1 in **A** CSCs and adjacent NCSE and **B** CA and adjacent NCGE by IHC staining (original magnification 20× and 40×). **C**, **D** The expression of Sp1 in CSCs and CAs compared to adjacent normal tissues. ***P < 0.001

The protein levels of Sp1 in human cervical squamous carcinoma, cervical adenocarcinoma, and para-carcinoma tissues were investigated. The pathological characteristics of cancer tissues and para-carcinoma tissues were analysed by H&E staining (Fig. 1A and B). Immunohistochemical (IHC) staining showed that the Sp1-positive score was higher in cervical squamous carcinoma and cervical adenocarcinoma tissues than in paracarcinoma tissues (Fig. 1C and D). This result suggests that Sp1 overexpression in cervical cancer tissues might be related to cervical tumorigenesis.

### Sp1 affects the biological behaviour of cervical cancer cells

**Fig. 2:**
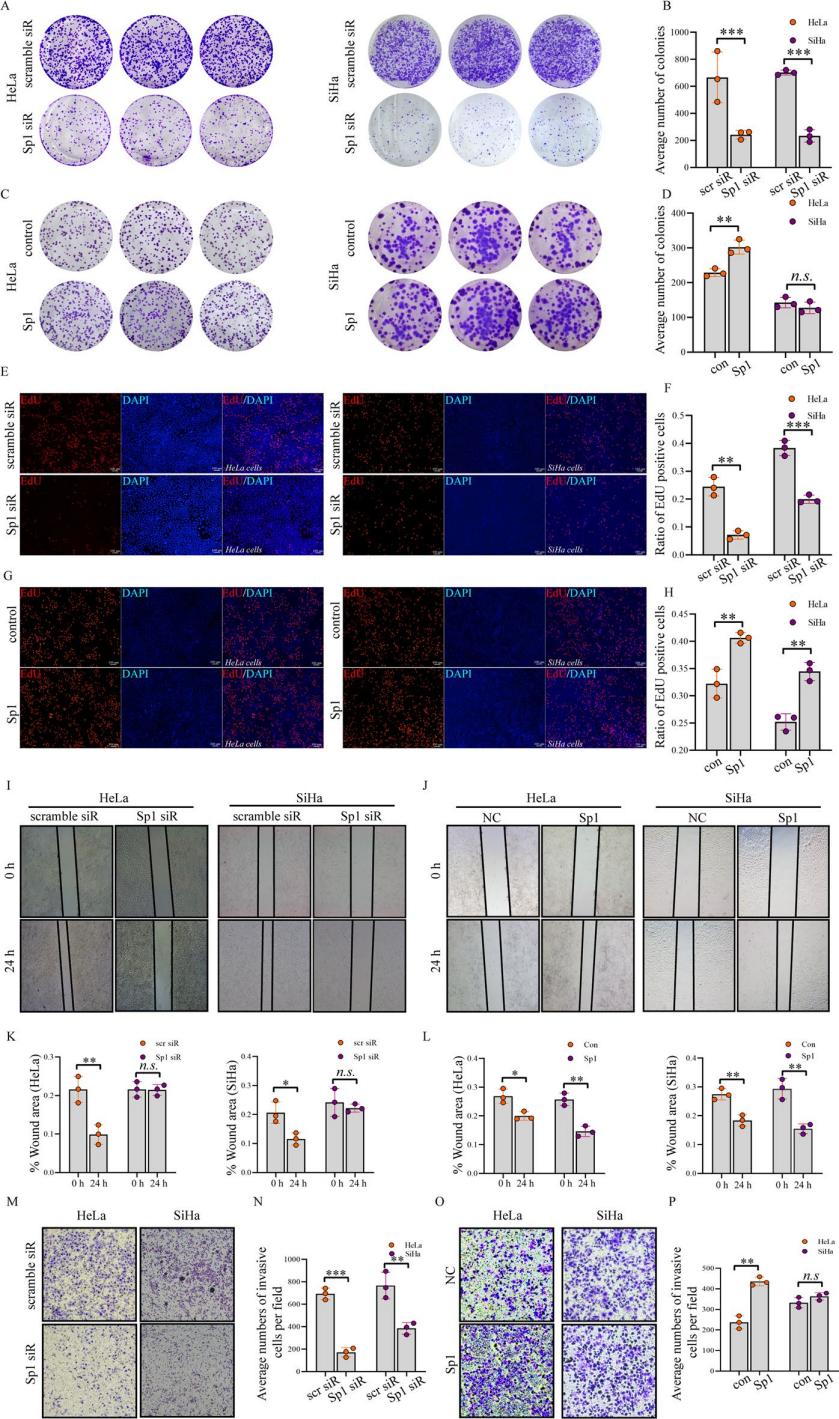
Sp1 promoted cell proliferation, invasion, and migration in cervical cancer cells. Cervical cancer cell lines were treated as indicated. The cell proliferation ability was detected by **A**–**D** colony formation assays and **E**–**H** EdU assays. The migration ability was determined by **I**–**L** wound healing assay. The migration and invasion abilities were detected by **M**–**P** transwell assay (Original magnification 10×). All experiments were repeated triplicate individually.*P < 0.05, **P < 0.01, ***P < 0.001, N. S *P* > 0.05

To verify the role of Sp1 during human cervical cancer tumorigenesis, Sp1 knockdown or overexpression on the biological behaviour in HeLa and SiHa cells was investigated. Cell proliferation was determined by a colony formation assay. We found that Sp1 knockdown reduced the number of colonies (Fig. 2A and B). Conversely, the number of colonies was increased by Sp1 overexpression (Fig. 2C and D). These data suggested that Sp1 was positively related to cell proliferation. To confirm this effect, cell proliferation was further analysed by EdU. The number of EdU-positive cells was decreased after Sp1 downregulation (Fig. 2E and F). In contrast, the EdU-positive cells were increased after Sp1 overexpression (Fig. 2G and H).

We next investigated whether Sp1 could affect cervical cancer cell migration. We found that Sp1 knockdown reduced cell migration (Fig. 2I and K), while its overexpression promoted cell migration (Fig. 2J and L). Furthermore, cell invasion was analysed by transwell assay. We found that Sp1 knockdown reduced cell invasion both in HeLa and SiHa cells (Fig. 2M and N), whereas Sp1 overexpression facilitated cell invasion in HeLa cells, but no significant difference was observed in SiHa cells (Fig. 2O and P). Altogether, these data suggest that Sp1 positively regulates the biological behaviour of cervical cancer cells.

### Sp1 downregulation promoted apoptosis and mitochondrial depolarization in cervical cancer cells

**Fig. 3:**
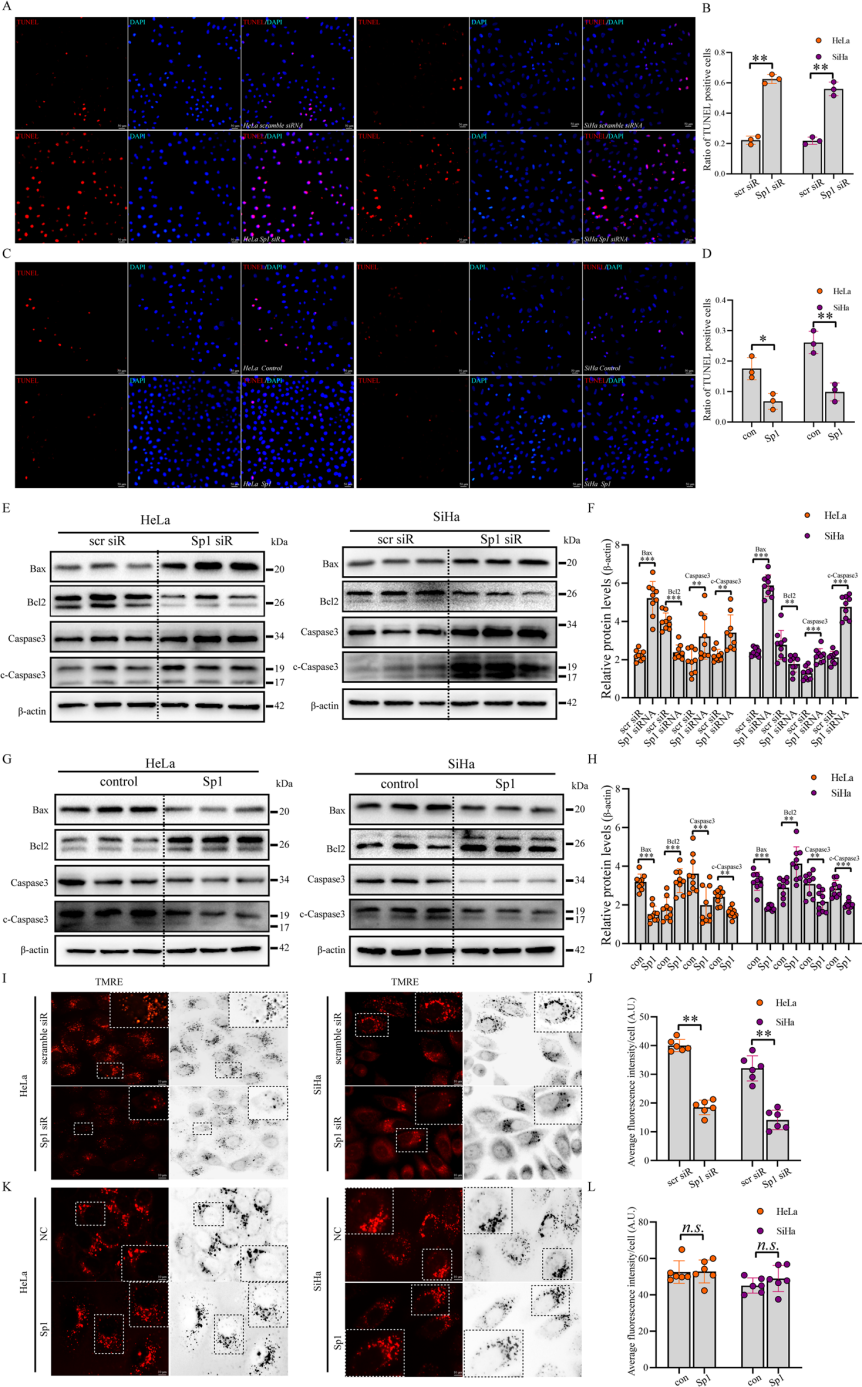
Sp1 modulated apoptosis and MMP of cervical cancer cells. **A**–**D** TUNEL (red) and DAPI (blue) staining of cervical cancer cells (original magnification 10×). **E**–**H** Western blot analysis was conducted to explore the levels of apoptosis-related proteins in cervical cancer cells treated as indicated. **I**–**L** MMP detection was performed in cervical cancer cells treated as indicated. All experiments were repeated triplicate individually.*P < 0.05, **P < 0.01, ***P < 0.001, N. S *P* > 0.05

To investigate the role of Sp1 in cell apoptosis, we conducted a TUNEL assay to examine the effect of Sp1 on cell apoptosis. As shown in (Fig. 3A–D), Sp1 downregulation increased the number of TUNEL-positive cells but was reduced by Sp1 overexpression. To strengthen these data, we analysed the levels of apoptosis-related proteins. We found that Bcl2 was decreased, but Caspase3, cleaved (c)-Caspase3, and Bax were upregulated after Sp1 knockdown (Fig. 3E and F). In contrast, Bcl2 was increased, but Caspase3, cleaved (c)-Caspase3, and Bax were downregulated after Sp1 overexpression (Fig. 3G and H). We next explored the level of MMP using tetramethylrhodamine ethyl ester (TMRE) after Sp1 knockdown or overexpression. TMRE is a mitochondrial dye that accumulates in active mitochondria due to its membrane potential. Depolarized mitochondria had decreased membrane potential and failed to sequester TMRE. We observed that Sp1 knockdown reduced the number of TMRE-positive mitochondria, but no significant difference was found by Sp1 overexpression when compared with the control group (Fig. 3I–L). These results suggested that Sp1 might be involved in maintaining MMP, and its downregulation might be correlated with mitochondrial depolarization, oxidative phosphorylation dysregulation, and apoptosis in cervical cancer cells. In summary, these data strongly implied that Sp1 might suppress apoptosis in cervical cancer cells.

### Sp1 enhanced the aerobic glycolysis activity in cervical cancer cells

**Fig. 4:**
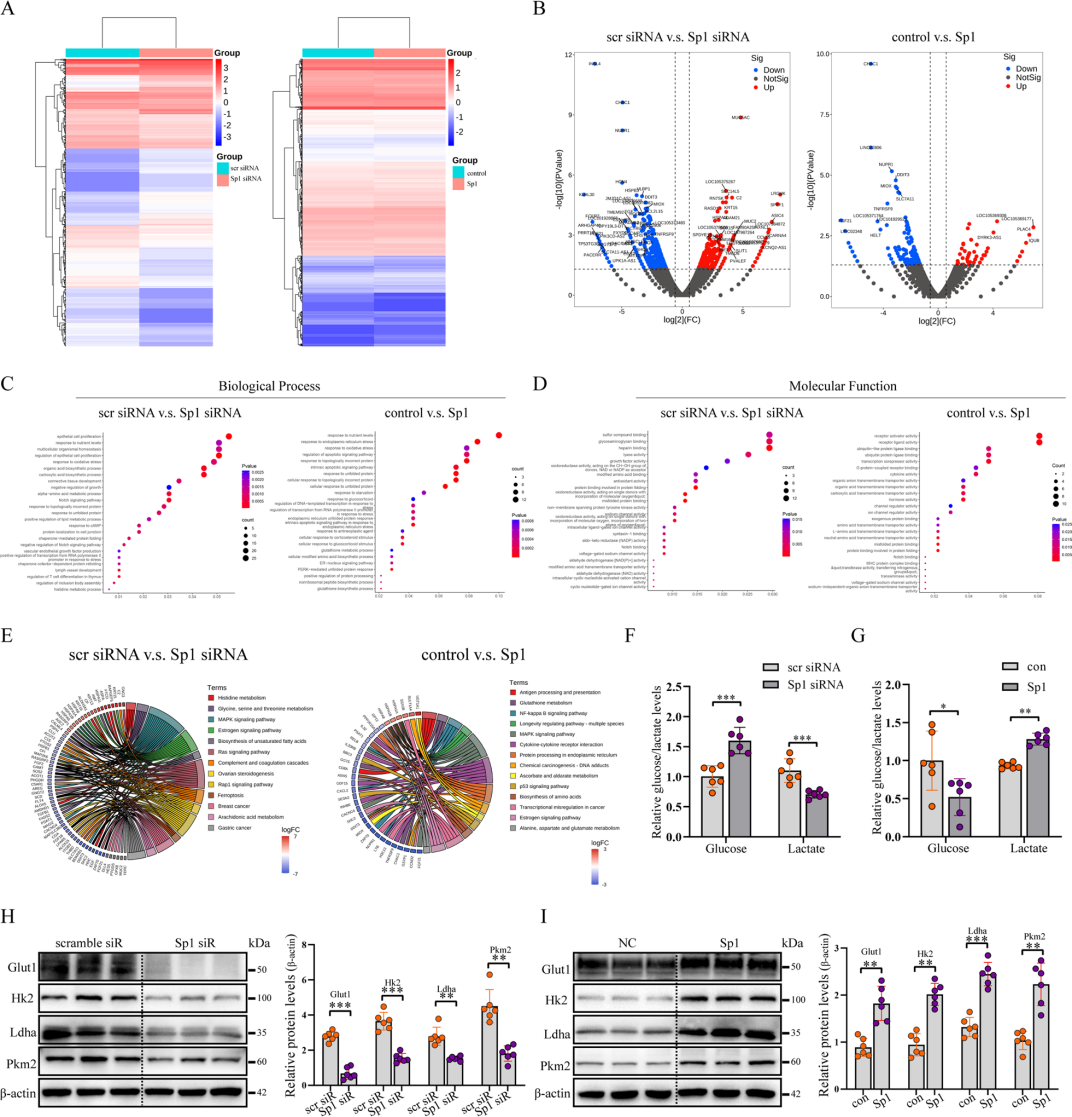
Sp1 enhanced aerobic glycolysis activity in cervical cancer cells. **A** DEG expression between the scramble siRNA group and Sp1 siRNA group (left) and DEG expression between the scramble control group and Sp1 group (right). **B** DEGs between the siRNA group and the Sp1 siRNA group (left) and DEGs between the control group and the Sp1 group (right). **C** GO analysis of DEGs by biological process. Left panel, scramble siRNA V.S. Sp1 siRNA. Right panel, control Group V.S. Sp1 group. **D** GO analysis of DEGs by molecular function. Left panel, scramble siRNA V.S. Sp1 siRNA. Right panel, control Group V.S. Sp1 group. Sp1 siR, siRNA against Sp1; scramble siR, control siRNA, control, empty vector, Sp1, vector encoding Sp1. **E** KEGG signalling pathway analysis of the DEGs. Left panel, scramble siRNA V.S. Sp1 siRNA. Right panel, control Group V.S. Sp1 group. **F** and **G** The relative levels of glucose and lactate in cervical cancer cells were treated as indicated. **H** and **I** Western blot analysis to detect glycolytic gene expression in cervical cancer cells treated as indicated. All experiments were repeated triplicate individually.*P < 0.05, **P < 0.01, ***P < 0.001

To uncover the mechanisms by which Sp1 affects cervical tumorigenesis, we performed mRNA sequencing and identified differentially expressed genes (DEGs) (Fig. 4A). The volcano plot shows that Sp1 knockdown yielded a higher number of differentially expressed genes than Sp1 overexpression (Fig. 4B). We next performed Gene Ontology (GO) biological process enrichment analysis and found that genes related to oxidative stress, regulation of growth, and apoptosis were affected by Sp1 knockdown or overexpression (Fig. 4C). Furthermore, GO molecular function enrichment analysis showed that genes related to the activity of oxidoreductase, antioxidants, NAD, and multiple receptors or ligases were affected by Sp1 knockdown or overexpression (Fig. 4D). The KEGG signalling pathway enrichment circos plot showed that DEGs were enriched in MAPK, oestrogen, and Ras signalling pathways after Sp1 knockdown or overexpression, which were demonstrated to be closely related to tumorigenesis and cell metabolism [28–30] (Fig. 4E). Previous studies have shown that metabolic abnormalities in tumour cells are typically manifested by increased aerobic glycolysis [31, 32]. To analyse the effect of Sp1 on aerobic glycolysis, we investigated glucose consumption and lactate production in HeLa cells after Sp1 knockdown or overexpression. As shown in Fig. 4F, the level of glucose was increased, while the level of lactate was decreased by Sp1 knockdown. In contrast, HeLa cells overexpressing Sp1 exhibited the opposite effects (Fig. 4G). Next, we examined the effects of the Sp1 level on the abundance of the key proteins on glycometabolism. Our data showed that (Fig. 4H and I) the protein levels of GLUT1, HK2, LDHA, and PKM2 were reduced after Sp1 downregulation but were upregulated by Sp1 overexpression. Taken together, these data indicated that the activity of aerobic glycolysis was positively regulated by Sp1 in cervical cancer cells.

### Sp1 promoted mitochondrial morphology remodelling by regulating mitochondrial dynamics-related proteins

**Fig. 5:**
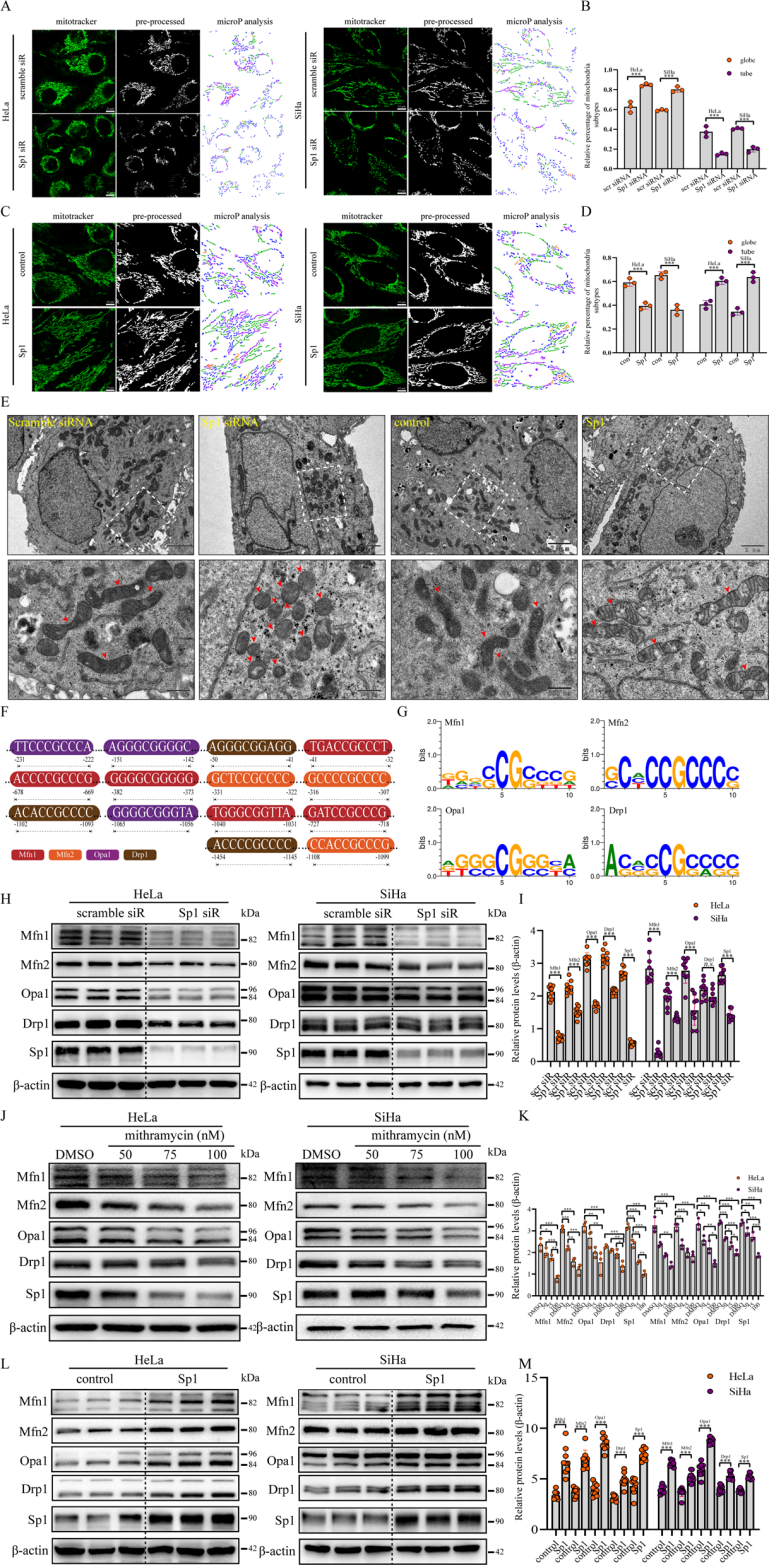
Sp1 promoted mitochondrial morphology remodelling. **A**–**D** The proportion of globe and tube mitochondria of cervical cancer cells with treatment as indicated, detected by MicroP (Original magnification: 63×). **E** Mitochondrial network in cervical cancer cells treated as indicated. Arrows indicate intermediate (mid) and fragmented mitochondria. **F** Sp1-binding sites on the Mfn1/2, Drp1, and Opa1 promoters and **G** the Sp1 consensus sequence via the UCSC genome browser. **H**–**M** The levels of Mfn1/2, Drp1, and Opa1 proteins in cervical cancer cells with Sp1 knockout or a specific inhibitor (mithramycin) and overexpression, as determined by Western blotting. All experiments were repeated triplicate individually.*P < 0.05, **P < 0.01, ***P < 0.001, N. S *P* > 0.05

To investigate whether Sp1 could affect mitochondrial morphological remodelling in cervical cancer cells, we analysed the morphological characteristics of mitochondria using microP [33]. Based on the morphology of the mitochondria, microP could divide the mitochondria into the following subtypes: small globe, straight branching tube, donut, twisting tube, and simple tube. In this study, we simplified these categories to globe or tube, which represented fragmented or fused mitochondria. As shown in Fig. 5A and B, the percentage of globe-shaped mitochondria was increased, but the percentage of tube-shaped mitochondria was reduced by Sp1 knockdown in both HeLa and SiHa cells. In contrast, the percentage of globe-shaped mitochondria was decreased, but the percentage of tube-shaped mitochondria was increased by Sp1 overexpression in HeLa and SiHa cells (Fig. 5C and D). In addition, we analysed the average areas of the mitochondrial network, but no significant difference was observed by Sp1 knockdown or overexpression (Additional file 1: Fig. S1A and B). We next performed an ultrastructural analysis to validate this morphological remodelling. Similar to the data shown above, the number of globe-shaped mitochondria was increased after Sp1 knockdown, but the number of tube-shaped mitochondria was increased by Sp1 overexpression (Fig. 5E and Additional file 1: Fig. S1C). We then searched for the potential Sp1-binding sites at the promoters of Mfn1/2, Drp1, and Opa1 and identified five Sp1-binding sites at the huMfn1 gene and three Sp1-binding sites at the huMfn2, huOpa1, and Drp1 genes from the UCSC genome browser (Fig. 5F and G). To explore the mechanisms of Sp1-mediated mitochondrial morphological remodelling in cervical cancer cells, the protein levels of mitochondrial dynamics-related proteins were determined by western blotting in cervical cancer cells with Sp1 knockdown or overexpression. We found that Sp1 knockdown (Fig. 5H and I) led to a decrease in the protein levels of Mfn1/2, Opa1, and Drp1 to different degrees. We then used a specific inhibitor of Sp1, mithramycin, to verify this result. We found that the protein levels of Mfn1/2, Opa1 and Drp1 were also reduced after mithramycin treatment (Fig. 5J and K). In contrast, the protein levels of Mfn1/2, Opa1, and Drp1 were higher after Sp1 overexpression (Fig. 5L and M). In addition, RNA sequencing data showed that Sp1 expression was positively correlated with the expression of Mfn1/2, Opa1, and Drp1 in tissues of cervical cancer patients (Additional file 1: Fig. S1D). Collectively, these data indicated that Sp1 promoted mitochondrial morphology remodelling by regulating both mitofusin and mitofission in cervical cancer cells.

### Sp1 overexpression contributed to xenograft tumour growth in vivo

**Fig. 6:**
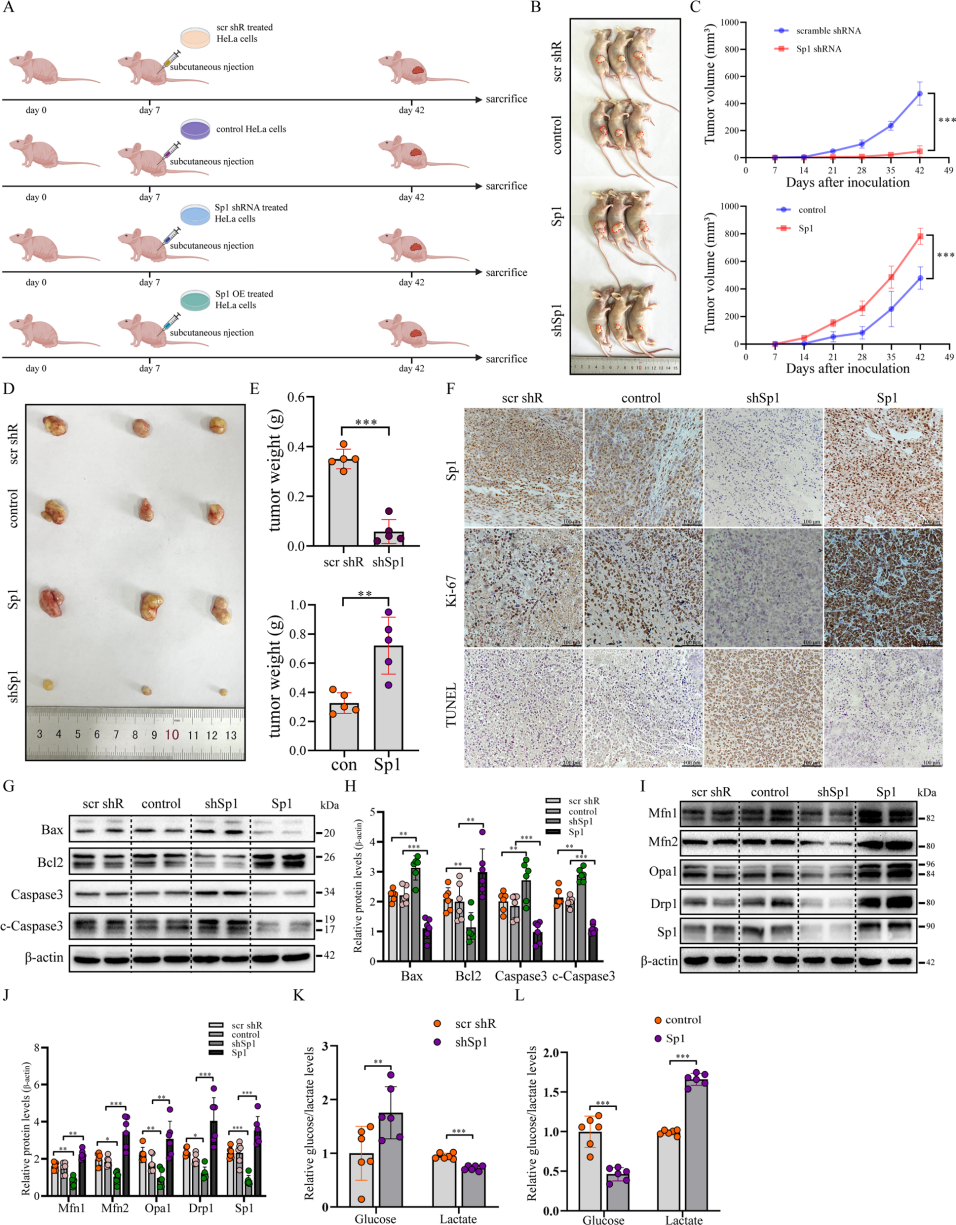
Sp1 enhanced subcutaneous tumour growth in vivo. **A** Experimental procedures for producing nude mouse models of subcutaneous tumorigenesis. **B** and **D** Subcutaneous nude mouse models and xenograft tumours. **C** and **E** The weight and volume of xenograft tumours. **F** IHC images of Sp1, Ki-67, and TUNEL assays in xenograft tumours of the Sp1-overexpression or Sp1-knockout groups. **G** and **H** The protein levels of apoptosis-related proteins were evaluated by Western blot analysis in HeLa subcutaneous xenografts with Sp1 overexpression or Sp1 knockout. **I** and **J** Western blot analysis of the levels of Mfn1/2, Drp1, and Opa1 in HeLa subcutaneous xenografts from the Sp1-overexpression or Sp1-knockout groups. **K** and **L** Relative glucose and lactate levels were determined in HeLa subcutaneous xenografts from the Sp1-overexpressing or Sp1-knockout groups. *P < 0.05, **P < 0.01, ***P < 0.001

To further verify the role of Sp1 in cervical tumorigenesis in vivo, Sp1-KD or Sp1-OE HeLa cells were injected subcutaneously into female nude mice (Fig. 6A). As shown in Fig. 6B–E, both the weight and the volume of the tumours were increased after Sp1 overexpression but were reduced by Sp1 knockdown when compared with the control group. Immunohistochemical (IHC) staining verified that the protein level of Sp1 was decreased in the Sp1-KD group but was increased in the Sp1-OE group (Fig. 6F). Importantly, there was a strong upregulation of Ki-67 in the Sp1-OE group but was decreased in the Sp1-KD nude mouse group, suggesting that cervical cancer cell proliferation was positively regulated by Sp1 (Fig. 6F). Furthermore, the TUNEL assay showed that the ratio of TUNEL-positive cells was upregulated in the Sp1-KD group but was reduced in the Sp1-OE group (Fig. 6F). To confirm this conclusion, we performed western blotting to analyse the levels of apoptosis-related proteins in tissues collected from different groups. We found that the protein levels of Bax, Caspase3, and cleaved (c)-Caspase3 were increased, but the level of Bcl2 was reduced in the Sp1-KD group compared with the control (Fig. 6G and H). In contrast, the Sp1-OE group exhibited the opposite effects (Fig. 6G and H). These data indicated that Sp1 played an antiapoptotic role in cervical tumorigenesis.

Based on the above data, mitochondrial dynamics were found to be regulated by Sp1. Therefore, we analysed the levels of mitochondrial dynamics-related proteins in different groups. We discovered that the levels of Mfn1/2, Drp1, and Opa1 were high in tissues from the Sp1-OE group but were reduced in tissues from the Sp1-KD group (Fig. 6I and J). These data further demonstrated that these mitochondrial dynamics-related proteins were targets of the transcription factor Sp1 in cervical cancer tissues. Finally, we investigated the effect of Sp1 dysregulation on the contents of glucose and lactate in vivo. In accordance with the results shown in the in vitro study, the relative level of glucose was increased in the Sp1-KD group but was reduced in the Sp1-OE group (Fig. 6K and L). Conversely, the relative content of lactate was decreased in the Sp1-KD group but was increased in the Sp1-OE group (Fig. 6K and L). Collectively, these data indicate that Sp1 could promote tumorigenesis by increasing aerobic glycolysis and breaking the balance between mitofusin and mitofission both in vitro and in vivo. Although the precise mechanism by which mitochondrial dynamics exert their effects on the activity of aerobic glycolysis remains to be elucidated, our study provides new insight into the regulatory role of Sp1 in cervical tumorigenesis and suggests that Sp1 may be an efficient target for treatment.

## Discussion

Sp1 can either activate or repress the conversion of normal cells into cancerous cells, thereby promoting or inhibiting cancer progression [34, 35]. Previous studies have reported that Sp1 is overexpressed in several cancers [36], including gastric cancer [37, 38], colon cancer [39, 40], lung cancer [41] and breast cancer [42], and is positively correlated with tumour progression. For example, Liu et al. demonstrated that Sp1 plays a key role in promoting proliferation rate, migration activity, and development of chemotherapy resistance in epithelial ovarian cancer [43]. Moreover, it was shown that Sp1 promoted proliferation and inhibit apoptosis in lung cancer cells [44, 45]. Although Wang et al. found that Sp1 was overexpressed in cervical cancer tissues [46], the regulatory mechanism of Sp1 in human cervical cancer remains to be elucidated. In this study, we found that Sp1 was overexpressed both in human cervical squamous carcinoma and cervical adenocarcinoma tissues. Inhibition of Sp1 suppressed cervical cell proliferation, migration, and invasion abilities, but these characteristics were restored by Sp1 overexpression in vitro. In vivo studies further demonstrated that the progression of cervical cancer was positively correlated with Sp1 levels.

In malignancies, the dysfunction of cell apoptosis plays a crucial role, which involves the activation of antiapoptotic proteins and inhibition of apoptotic proteins. Chuang et al. reported that Sp1 overexpression led to apoptosis [47]; however, an increasing number of studies showed that Sp1 knockdown induced apoptosis in cervical cancer cells [18]. In this work, we demonstrated that cell apoptosis was increased after Sp1 knockdown but was decreased by Sp1 overexpression. In vivo data confirmed that Sp1 negatively regulated cell apoptosis in cervical cancer. The mitochondrial membrane potential (MMP) is pivotal for energy storage during oxidative phosphorylation [48]. Furthermore, mitochondrial depolarization was regularly observed during cell apoptosis [49]. In non-quenching mode, we found that MMP was decreased by Sp1 knockdown in cervical cancer cells. Collectively, these findings indicate that Sp1 could promote cervical tumorigenesis by improving cell proliferation, migration, and invasion and inhibiting apoptosis.

To investigate the mechanisms of Sp1 in regulating cervical tumorigenesis, we performed mRNA sequencing and found that genes related to cell metabolism were significantly changed by Sp1 knockdown or overexpression. Sp1 was found to play a role in tumour cell metabolism. For instance, cAMP and endothelin-1 can cooperate with Sp1 to promote GLUT1 transcription [50]. In addition, hyperactive aerobic glycolysis has been well established as the major metabolic phenotype in cervical cancer and other malignancies [51, 52]. Thus, we suggest that Sp1 might regulate cervical tumorigenesis by regulating aerobic glycolysis in cervical cancer cells. To confirm this, we found that proteins related to aerobic glycolysis as well as the level of glucose were decreased after Sp1 knockdown but were increased by Sp1 overexpression both in vitro and in vivo. Based on the above results, we concluded that Sp1 positively regulates aerobic glycolysis in cervical cancer.

Mitochondria are the “energy factories” in cells and coordinate physiology and metabolism. Mitochondrial dynamics are indispensable for maintaining mitochondrial quality [8]. Dysregulation of mitofusin or mitofission has been observed in different types of cancers and was demonstrated to be correlated with tumorigenesis [8, 9, 53]. Mitochondrial fission is an early event of apoptosis and plays a critical role in cell apoptosis. And excessive mitochondrial fission appears to be a requisite step in intrinsic apoptosis pathways [54]. An increasing number of studies have also found that mitochondrial dynamics could affect the activity of aerobic glycolysis in cancer cells [14, 55]. For example, Li et al. reported that a higher number of fragmented mitochondria associated with enhanced expression of mitofission-related proteins, such as Drp1, may correlate with impaired apoptosis and increased glycolysis activity [56, 57]. In the present study, we investigated whether Sp1 could affect mitochondrial morphology in cervical cancer cells. Our data showed that mitochondrial elongation was increased after Sp1 overexpression, but mitochondrial fragmentation was enhanced by Sp1 knockdown. Mechanistically, we found that Sp1 could facilitate mitochondrial remodelling by promoting the mitofusin-related proteins Mfn1/2 and Opa1 and the mitofission-related protein Drp1 both in vivo and in vitro. These results suggested that Sp1 overexpression rebuilt the balance between mitofusin and mitofission and was more likely to improve mitofusin than mitofission during cervical tumorigenesis. Although the percentage of elongated mitochondria was increased, we showed that apoptosis was reduced by Sp1 overexpression. It may be due to the complicated relationships between apoptosis and mitochondrial morphology, which are still obscure. Importantly, Thibaud et al. reported that the mobilization of Bax, which is regulated by the remodeling of mitochondrial shape, instead of mitofission per se, could efficiently induce apoptosis [58]. Further work need be done to uncover the intrinsic interaction between mitochondrial dynamics and apoptosis after Sp1 overexpression.

## Conclusions

**Fig. 7:**
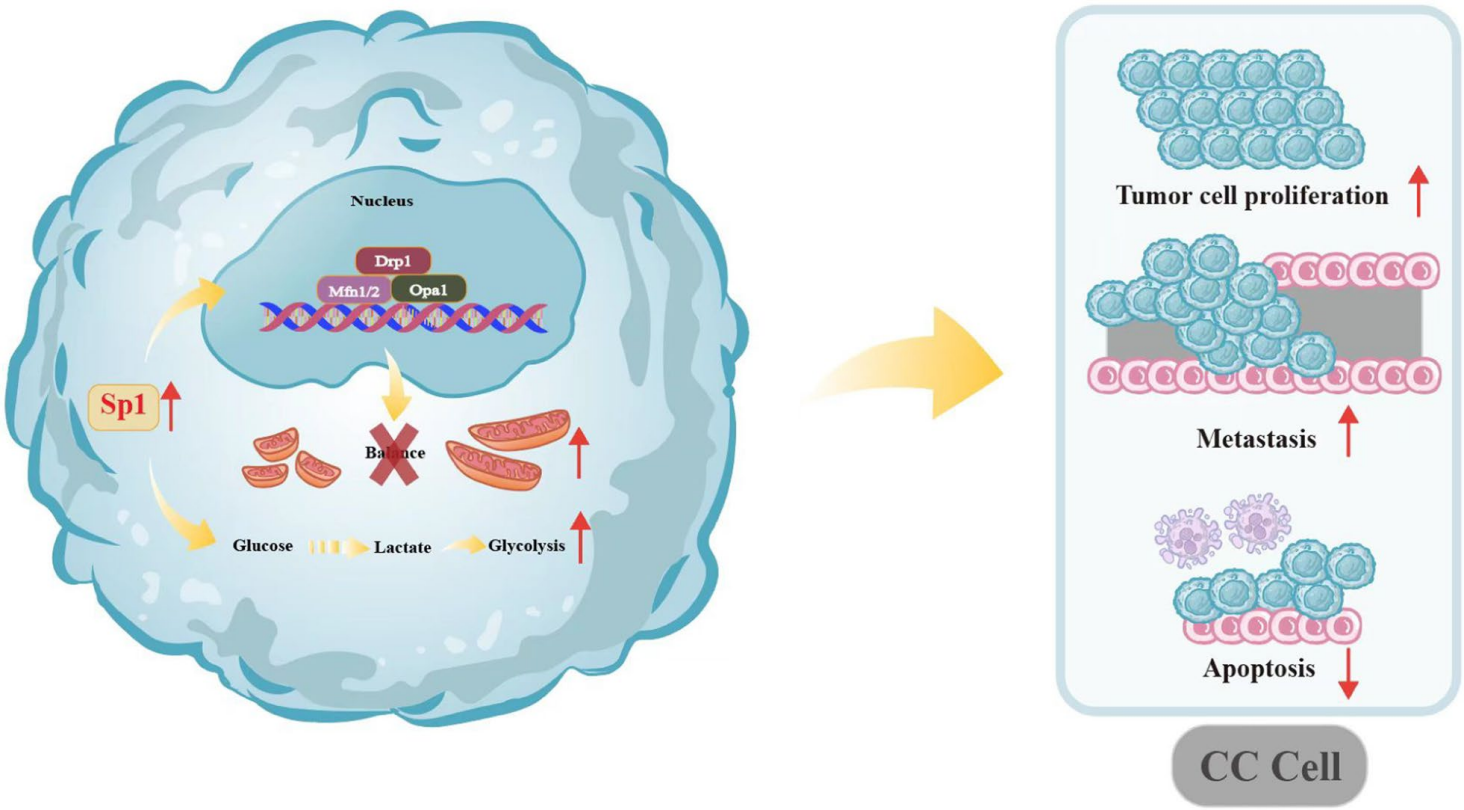
Sp1 promoted the tumorigenesis of cervical cancer. Sp1 is involved in promoting cell proliferation, migration, invasion, and tumour growth and inhibiting cell apoptosis in cervical cancer. Sp1 boosts the tumorigenesis of cervical cancer by reducing aerobic glycolysis and promoting mitochondrial remodelling by rebuilding the balance between mitofusin and mitofission, and then it promotes cell growth and inhibits tumour cell apoptosis in cervical cancer

In this study, we investigated the potential mechanisms of Sp1 in cervical tumorigenesis and found that overexpression of Sp1 promotes the proliferation, migration, and invasion and reduces apoptosis of human cervical cancer cells in vitro and in vivo by improving aerobic glycolysis and promoting mitochondrial remodelling by rebuilding the balance between mitofusin and mitofission (Fig. 7). These findings provide novel insights into the role of Sp1 in cervical tumorigenesis, and Sp1 may be a potential therapeutic target for cervical cancer treatment.

## Supplementary Information

**Additional file 1:** **Figure S1.** Relationship between Sp1 and mitochondrial network. (A-B) The mitochondrial morphology of Sp1-knockout and overexpression cervical cancer cells by Tomm70A staining. (C) The proportion of globe and tube mitochondria of cervical cancer cells with treatment as indicated, detected by electron microscopy. (D) The correlation between mitochondrial dynamics-related proteins and Sp1 expression. The expression of Sp1 was positively related to Mfn1/2, Drp1, and Opa1.

## Data Availability

The datasets used and/or analyzed during the current study are available from the corresponding author upon reasonable request.

## Abbreviations

Sp1: Specificity protein 1
MFN1: Mitochondrial fusion protein 1
MFN2: Mitochondrial fusion protein 2
DRP1: Dynamin-related protein 1
Opa1: Optic atrophy1
ATP: Adenosine-triphosphate
SP/KLF: Specificity protein/Krüppel-like factor
H&E: Hematoxylin and eosin
IHC: Immunohistochemistry
EdU: 5-Ethynyl-20-deoxyuridine
TUNEL: Terminal deoxynucleotidyltransferase-mediated dUTP-biotin nick end labeling
Bcl2: B-cell lymphoma-2
Bax: BCL2-associated X protein
MMP: Mitochondrial membrane potential
TMRE: Tetramethylrhodamine ethyl ester
mRNA: Messenger RNA
DEGs: Different expression genes
GO: Gene Ontology
NAD: Nicotinamide adenine dinucleotide
KEGG: Kyoto Encyclopedia of Genes and Genomes
MAPK: Mitogen-activated protein kinase
GLUT1: Glucose transporter type 1
HK2: Hexokinase 2
LDHA: Lactate dehydrogenase A
PKM2: Pyruvate kinase isoform M2
UCSC: University of California Santa Cruz
KD: Knockdown
OE: Overexpression
DAB: 3, 5-Diaminobenzidine
siRNA: Small interfering RNA
MOI: Multiplicity of infection
GFP: Green Fluorescent Protein
CCK-8: Cell Counting Kit-8
OD: Optical density
