# Supplementary figures and images for: Sp1 promotes tumour progression by remodelling the mitochondrial network in cervical cancer
Source: J Transl Med. 2023 May 6;21:307. doi: 10.1186/s12967-023-04141-3 (PMC10163764; doi:10.1186/s12967-023-04141-3)

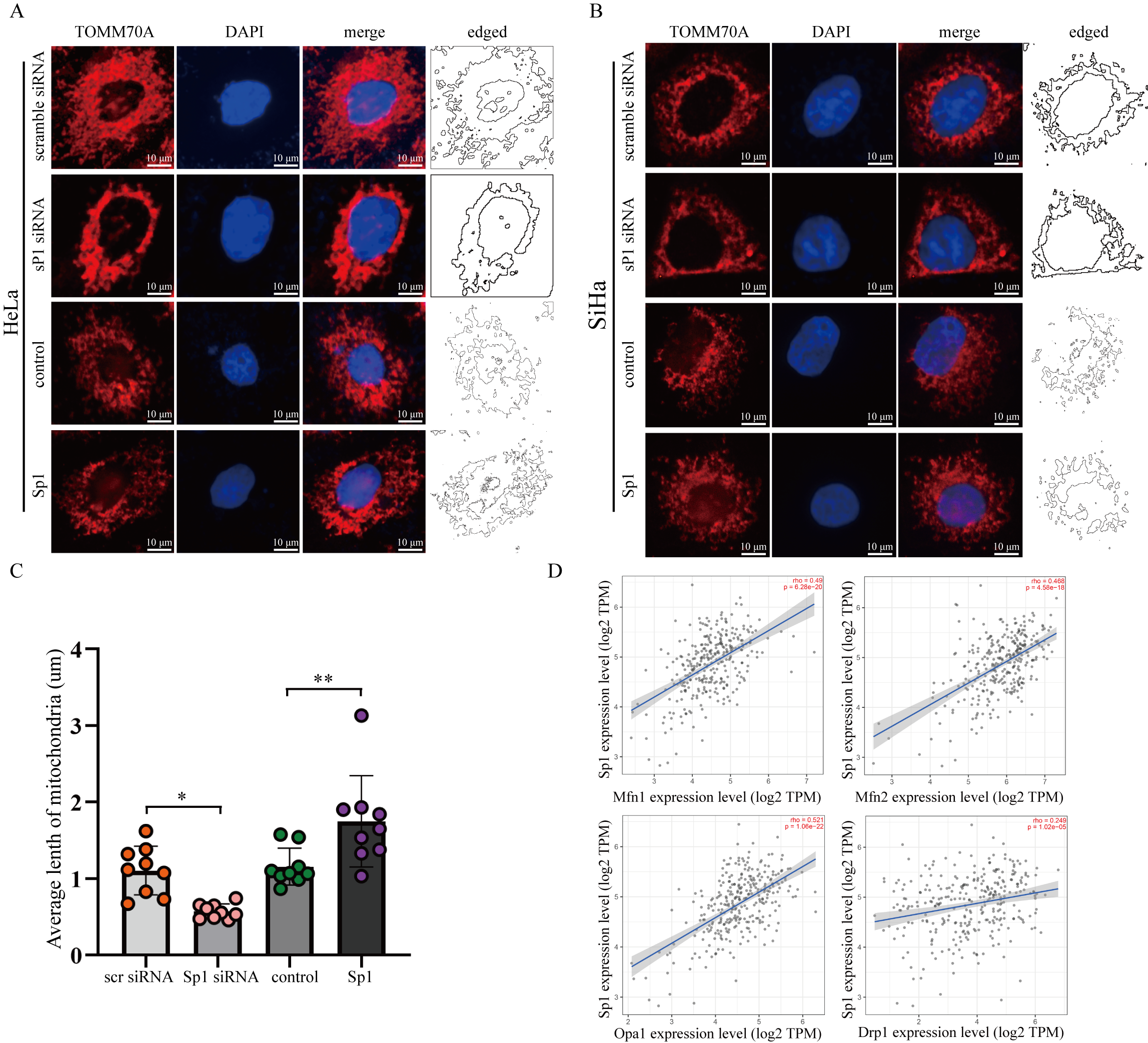

Supplement: Supplementary file 1 — Additional file 1: Figure S1. Relationship between Sp1 and mitochondrial network. (A-B) The mitochondrial morphology of Sp1-knockout and overexpression cervical cancer cells by Tomm70A staining. (C) The proportion of globe and tube mitochondria of cervical cancer cells with treatment as indicated, detected by electron microscopy. (D) The correlation between mitochondrial dynamics-related proteins and Sp1 expression. The expression of Sp1 was positively related to Mfn1/2, Drp1, and Opa1. [file 12967_2023_4141_MOESM1_ESM.tif]
